# 2281 Prophylactic broad-spectrum antibiotics for childhood malnutrition

**DOI:** 10.1017/cts.2018.179

**Published:** 2018-11-21

**Authors:** Amy Elizabeth Langdon, Xiaoqing Sun, Sheila Isanaka, Gautam Dantas

**Affiliations:** 1 Institute of Clinical and Translational Sciences, Washington University in St. Louis; 2 Washington University in St. Louis; 3 Medecins Sans Frontiers, Epicentre, France

## Abstract

OBJECTIVES/SPECIFIC AIMS: A course of oral broad-spectrum antibiotics frequently has a positive effect on morbidity and mortality in severe acute malnutrition (SAM), but the actual mechanism for this effect is unknown. This mechanism is especially important to find and quantify because of the possibility that using antibiotics prophylactically may accelerate the danger from antibiotic resistant infections. This study aims to answer (1) how antibiotic therapy improves the nutritional recovery and (2) how much it affects the prevalence of resistance genes in the microbiome. METHODS/STUDY POPULATION: Stool samples were collected from children with SAM between 6 and 60 weeks of age who received either one week of amoxicillin or placebo (n=164). The children were followed for 12 weeks with longitudinal sampling, and a subset were followed out to 2 years. All samples were frozen at −80°C and prepared for metagenome shotgun sequencing via the Illumina Nextera platform. RESULTS/ANTICIPATED RESULTS: Antibiotic treatment at the start of the nutritional program is associated with significant improvements in weight gain, mid-upper-arm circumference, and graduation from the treatment program. It is also associated with qualitative decreases in early-life fermenter Lactobacillus and known enteropathogen Campylobacter. Two years after the use of amoxicillin, the Shannon diversity index is significantly higher than that of malnourished children (effect size 0.507, 95% CI: 0.204–0.630, *p*=0.0007), while children who received placebo are not distinguishable from malnourished children by the same metric (effect size 0.147, 95% CI: −0.311, 0.630, *p*=0.5878). Sustained antibiotic resistance gene enrichment within the microbiota did not occur, as the enrichment effects disappears by week 4 of follow-up. DISCUSSION/SIGNIFICANCE OF IMPACT: The use of amoxicillin to treat uncomplicated SAM has therapeutic benefits visible by anthropometry and by content of the gut microbiota. The main concern with the use of prophylactic antibiotics for this purpose is the effect on antibiotic resistance gene enrichment in the children’s microbiota. This concern was not supported here. The benefit/cost ratio for the use of prophylactic antibiotics for individuals in this cohort is positive when weighing effects on anthropometry, microbiome, and antibiotic resistance. The results of this study impact the treatment of millions of children each year at nutritional therapy clinics around the world.

